# SARS-CoV-2 coinfections among pertussis cases identified through the Enhanced Pertussis Surveillance system in the United States, January 2020–February 2023

**DOI:** 10.1371/journal.pone.0311488

**Published:** 2024-12-04

**Authors:** Isha Berry, Matthew Cole, Benjamin Silk, Fiona P. Havers, Erin Youngkin, Adam Misiorski, Samantha Sefton, Yeng Vang, Emma Stanislawski, Suzanne McGuire, Noel Silhan, Tami H Skoff, Amy B. Rubis

**Affiliations:** 1 Division of Bacterial Diseases, National Center for Immunization and Respiratory Diseases, Centers for Disease Control and Prevention, Atlanta, Georgia, United States of America; 2 Epidemic Intelligence Service, Centers for Disease Control and Prevention, Atlanta, Georgia, United States of America; 3 Coronavirus and Other Respiratory Viruses Division, National Center for Immunization and Respiratory Diseases, Centers for Disease Control and Prevention, Atlanta, Georgia, United States of America; 4 Colorado Department of Public Health and Environment, Denver, Colorado, United States of America; 5 Connecticut Department of Public Health, Hartford, Connecticut, United States of America; 6 Georgia Emerging Infections Program, Atlanta, Georgia, United States of America; 7 Division of Infectious Diseases, Emory University School of Medicine, Atlanta, Georgia, United States of America; 8 Atlanta VA Medical Center, Decatur, Georgia, United States of America; 9 Minnesota Department of Public Health, St. Paul, Minnesota, United States of America; 10 New Mexico Department of Health, Santa Fe, New Mexico, United States of America; 11 New York State Department of Public Health, Albany, New York, United States of America; 12 Oregon Health Authority, Portland, Oregon, United States of America; Gabriele d’Annunzio University of Chieti and Pescara: Universita degli Studi Gabriele d’Annunzio Chieti Pescara, ITALY

## Abstract

**Background:**

Bacterial and viral respiratory coinfections are common, but the prevalence of SARS-CoV-2 infections among pertussis cases has not been estimated. We examine the prevalence and temporality of SARS-CoV-2 infections among pertussis patients and describe pertussis clinical severity among patients with and without SARS-CoV-2 coinfections.

**Methods:**

Confirmed and probable pertussis cases among individuals with cough onset between January 1, 2020 and February 15, 2023 were identified through surveillance in seven Enhanced Pertussis Surveillance (EPS) sites. Pertussis cases with a laboratory-confirmed SARS-CoV-2 infection detected within 30 days before or after pertussis cough onset were defined as coinfections. We describe patient demographics, symptoms, and severe complications and outcomes (seizures, encephalopathy, pneumonia, hospitalization, or death) by coinfection status.

**Results:**

Among 765 pertussis cases reported during the study period, the prevalence of SARS-CoV-2 coinfections was 0.78% [6/765]. Among the six patients meeting the coinfection definition, the majority (83.3% [5/6]) had SARS-CoV-2 infections detected following pertussis cough onset. Compared to those with no known coinfection, a higher proportion of those with coinfections reported severe complications or outcomes (50.0% [3/6] vs. 5.2% [36/694]).

**Discussion:**

Although the prevalence of pertussis patients with SARS-CoV-2 coinfections was low, patients with coinfections reported more severe complications and outcomes compared to those with pertussis alone. Given the decline in reported pertussis cases during the COVID-19 pandemic, continued monitoring of pertussis incidence alongside respiratory viral infections will be important as the pertussis burden returns to pre-pandemic levels.

## Introduction

Pertussis is a highly contagious, vaccine-preventable respiratory infection caused by the *Bordetella pertussis* bacterium. Clinical presentations of pertussis typically include prolonged cough, often characterized by a “whoop”, and may be accompanied by vomiting. However, symptoms can range from relatively mild cough to severe respiratory illness including pneumonia, seizures, encephalopathy, respiratory failure, and death [1, 2]. In the United States (U.S.), pertussis incidence has increased since the late 1980s with disease incidence peaking at 2–5-year intervals [3, 4]. Given the cyclic nature of pertussis, an increasing trend in reported disease could have been expected in 2020; however, this pattern was disrupted during the COVID-19 pandemic when there were notable declines in reported pertussis [3]. The number of reported cases dropped from over 18,000 in 2019 to approximately 6,000 in 2020 and preliminary data report fewer than 3,000 per year in 2021 and 2022 [3].

The first cases of SARS-CoV-2 were detected in the U.S. in January 2020. Similar to pertussis, SARS-CoV-2 infections can range from mild to severe illness, with serious manifestations including pneumonia, acute respiratory distress, and death [5]. Bacterial and viral respiratory coinfections can increase illness severity [6, 7]. Commonly reported bacterial coinfections with SARS-CoV-2 include *Mycoplasma pneumoniae*, *Pseudomonas aeruginosa*, *Staphylococcus aureus*, and *Haemophilus influenzae* [8]. While *Bordetella* spp. coinfections have also been reported [9, 10], little is known about the epidemiology and clinical severity of pertussis and SARS-CoV-2 coinfections.

In this descriptive study, we examine the prevalence and temporality of SARS-CoV-2 infections among pertussis patients reported through the Centers for Disease Control and Prevention’s (CDC) Enhanced Pertussis Surveillance (EPS) system and report demographic characteristics and pertussis clinical severity among patients with and without SARS-CoV-2 coinfections.

## Materials and methods

Confirmed and probable pertussis cases among individuals with cough onset between January 1, 2020 and February 15, 2023 were identified through EPS as part of the Emerging Infections Program Network (EIP) in seven states (Colorado: 5-county Denver area; Connecticut; Georgia: 20-county Atlanta area; Minnesota; New Mexico; New York: 15-county Rochester/Albany area; and Oregon: 3-county Portland area). Cases were classified according to the Council of State and Territorial Epidemiologists’ (CSTE) case definition [S1 Text] [11]. EPS uses a standardized case report form to collect demographic, clinical, vaccination, and epidemiologic information from patient and provider interviews, medical records, and state immunization registries. Patients aged two months through 20 years were considered up to date on pertussis-containing vaccines if they received all vaccines recommended for their age by the Advisory Committee on Immunization Practices (ACIP); patients aged ≥21 years were considered up to date if they had received ≥1 dose of Tdap. For this analysis, only vaccine doses administered ≥14 days prior to cough onset were considered valid.

Individuals with SARS-CoV-2 infections confirmed by PCR reported through the Nationally Notifiable Diseases Surveillance System (NNDSS) and COVID-19–associated hospitalizations reported through EIP’s COVID-19–associated Hospitalization Surveillance Network (COVID-NET) were identified. NNDSS and COVID-NET use standardized case report forms to collect demographic and clinical characteristics. Detection dates for SARS-CoV-2 infections were calculated as the earliest of available clinical dates (symptom onset, specimen collection) or, if missing, the initial date CDC received the case.

EPS site staff linked pertussis cases to laboratory-confirmed SARS-CoV-2 cases detected within one year before or after pertussis cough onset to ensure broad capture of patients for inclusion in the analysis. Temporality of SARS-CoV-2 infections among pertussis patients was examined graphically. Patients with coinfections were defined as those who met the CSTE pertussis case definition [11] and had a laboratory-confirmed SARS-CoV-2 infection detected ≤30 days before or after pertussis cough onset. A 30-day window is in line with previous literature [12], and enables robust capture of coinfections given the overlap in symptoms between SARS-CoV-2 and pertussis and the diagnostic challenges that could have occurred during this period. Patients with SARS-CoV-2 infections detected 31–365 days before or after pertussis cough onset were excluded [S1 Fig]. We describe demographics, symptoms, and severe complications and outcomes by coinfection status. We define severe complications and outcomes as seizures, encephalopathy, pneumonia, hospitalization, or death. Analyses were conducted in R version 4.1.2.

This activity was reviewed by CDC and was conducted consistent with applicable federal law and CDC policy. (See e.g., 45 C.F.R. part 46.102(l)(2), 21 C.F.R. part 56; 42 U.S.C. §241(d); 5 U.S.C. §552a; 44 U.S.C. §3501 et seq).

## Results

A total of 765 pertussis cases were reported through EPS during the study period, of which six met the coinfection definition for an overall coinfection prevalence of 0.78%. Of all patients, 90.7% [694/765] had pertussis alone, while 9.3% [71/765] had a SARS-CoV-2 infection detected within one year of pertussis cough onset [S1 Fig]. Of these patients, 59.1% [42/71] had SARS-CoV-2 infections detected 31–365 days (median: 246 days) *after* pertussis cough onset and 32.4% [23/71] had SARS-CoV-2 infections detected 31–365 days (median: 162 days) *before* pertussis cough onset and were excluded. Among the six patients meeting the coinfection definition, one had a SARS-CoV-2 infection detected on the same day as pertussis cough onset, two had SARS-CoV-2 infections detected ≤7 days following pertussis cough onset, and three had SARS-CoV-2 infections detected 8–30 days following pertussis cough onset [S2 Fig].

The ages of patients with coinfections ranged from <1–63 years, with a median of 41 years, while those with pertussis alone were <1–85 years with a median of 15 years. Among patients with coinfections, 50.0% [2/4] were Hispanic or Latino and 40.0% [2/5] were Black or African American persons; among those with pertussis alone, 19.4% [126/651] were Hispanic or Latino and 6.8% [44/649] were Black or African American persons [Table 1]. Among patients with and without coinfections, 40.0% [2/5] and 59.5% [340/571] were up to date on pertussis vaccines, respectively. COVID-19 vaccination status was not available for coinfected patients.

**Table 1:** Characteristics of pertussis patients by SARS-CoV-2 coinfection status, 2020–2023.

	Analytic Sample^a^, n (%)	Patients by Coinfection Status	
		Pertussis and SARS-CoV-2 Coinfections, n (%)	Pertussis Only, n (%)
Totals	N = 700	N = 6	N = 694
**Demographic & Epidemiologic Characteristics**			
**Sex** ^b^			
Male	312 (44.6)	3 (50.0)	309 (44.6)
Female	387 (55.4)	3 (50.0)	384 (55.4)
**Age (years)**			
<1	62 (8.9)	2 (33.3)	60 (8.6)
1–6	127 (18.1)	0 (0.0)	127 (18.3)
7–10	62 (8.9)	0 (0.0)	62 (8.9)
11–19	164 (23.4)	0 (0.0)	164 (23.6)
≥20	285 (40.7)	4 (66.7)	281 (40.5)
**Race** ^b^			
White persons	552 (84.4)	1 (20.0)	551 (84.9)
American Indian or Alaskan Native persons	9 (1.4)	0 (0.0)	9 (1.4)
Asian/Pacific Islander	17 (2.6)	0 (0.0)	17 (2.6)
Black or African American persons	46 (7.0)	2 (40.0)	44 (6.8)
People who identify as other race(s)	30 (4.6)	2 (40.0)	28 (4.3)
**Ethnicity** ^b^			
Hispanic or Latino persons	128 (19.5)	2 (50.0)	126 (19.4)
Non-Hispanic or non-Latino persons	527 (80.5)	2 (50.0)	525 (80.6)
**Pertussis Immunization** ^b^			
Up to date	342 (59.4)	2 (40.0)	340 (59.5)
Insufficiently vaccinated	63 (10.9)	1 (20.0)	62 (10.9)
Unvaccinated	171 (29.7)	2 (40.0)	169 (29.6)
Ineligible (too young)	7 (—)	0 (—)	7 (—)
**Pertussis Case Classification** ^c^			
Confirmed	391 (55.9)	5 (83.3)	386 (55.6)
Probable	309 (44.1)	1 (16.7)	308 (44.4)
**Symptoms**			
**Cough** ^b^			
Yes	699 (100.0)	6 (100.0)	693 (100.0)
No	0 (0.0)	0 (0.0)	0 (0.0)
**Paroxysmal Cough** ^b^			
Yes	617 (89.9)	1 (20.0)	616 (90.5)
No	69 (10.1)	4 (80.0)	65 (9.5)
**Whoop** ^b^			
Yes	263 (39.1)	2 (40.0)	261 (39.1)
No	410 (60.9)	3 (60.0)	407 (60.9)
**Post-tussive Vomiting** ^b^			
Yes	314 (45.5)	3 (50.0)	311 (45.5)
No	376 (54.5)	3 (50.0)	373 (54.5)
**Apnea** ^b^			
Yes	92 (13.5)	1 (25.0)	91 (13.4)
No	589 (86.5)	3 (75.0)	586 (86.6)
**Cyanosis** ^b^			
Yes	34 (5.1)	1 (20.0)	33 (4.9)
No	639 (94.9)	4 (80.0)	635 (95.1)
**Fever** ^b^			
Yes, patient and/or provider reported	129 (19.3)	1 (16.7)	128 (19.4)
No	538 (80.7)	5 (83.3)	533 (80.6)
**Severe Complications & Outcomes**			
**Any Severe Complication or Outcome** ^d^			
Yes	39 (5.6)	3 (50.0)	36 (5.2)
No	661 (94.4)	3 (50.0)	658 (94.8)
**Seizures** ^b^			
Yes	3 (0.4)	1 (16.7)	2 (0.3)
No	688 (99.6)	5 (83.3)	683 (99.7)
**Encephalopathy** ^b^			
Yes	3 (0.4)	0 (0.0)	3 (0.4)
No	686 (99.6)	5 (100.0)	681 (99.6)
**Pneumonia** ^b^			
Yes	24 (9.4)	1 (25.0)	23 (9.2)
No	230 (90.6)	3 (75.0)	227 (90.8)
Not tested	421 (—)	2 (—)	419 (—)
**Hospitalized** ^b^			
Yes	38 (5.5)	3 (50.0)	35 (5.1)
No	657 (94.5)	3 (50.0)	654 (94.9)
**Death**			
Yes	1 (0.1)	1 (16.7)	0 (0.0)
No	699 (99.9)	5 (83.3)	694 (100.0)

**Note**:

^a^Patients with SARS-CoV-2 infections detected 31–365 days before or after pertussis cough onset were excluded from all statistical analyses (n = 65).

^b^Calculated out of those with known and eligible responses. Unknown responses for sex n = 1 (0.1%); race n = 46 (6.6%); ethnicity n = 45 (6.4%); pertussis immunization n = 117 (16.7%); cough n = 1 (0.1%); paroxysmal cough n = 14 (2.0%); whoop n = 27 (3.8%); post-tussive vomiting n = 10 (1.4%); apnea n = 19 (2.7%); cyanosis n = 27 (3.9%); fever n = 33 (4.7%); seizures n = 9 (1.2%); encephalopathy n = 11 (1.5%); pneumonia n = 25 (3.6%); hospitalized n = 5 (0.7%).

^c^Cases were classified according to the Council of State and Territorial Epidemiologists case definition [11].

^d^Any severe complication or outcome was defined as reporting seizures, encephalopathy, pneumonia, hospitalization, or death.

All patients reported cough, as it is part of the pertussis case definition, but 20.0% [1/5] with coinfections and 90.5% [616/681] with pertussis alone reported paroxysmal cough. Table 1 details proportions of patients with and without coinfections reporting other symptoms including whoop, post-tussive vomiting, and cyanosis. While 50.0% [3/6] of patients with coinfections reported any severe complication or outcome including seizures, hospitalization, and death, only 5.2% [36/694] with pertussis alone reported any of these. The coinfected patients with severe complications or outcomes were aged <1, 60, and 63 years, and none were up to date on pertussis vaccines. Additional information on patients with coinfections is provided in S1 Table.

## Discussion

In this descriptive study of U.S. Enhanced Pertussis Surveillance data reported between January 2020 and February 2023, the prevalence of pertussis and SARS-CoV-2 coinfections was low. However, demographic characteristics and disease severity varied between patients with and without coinfections. The results of this small study suggest pertussis and SARS-CoV-2 coinfection is more severe than pertussis infection alone.

We may have only identified a low number of coinfections during the study period due to the fact that reported pertussis case counts were much lower in the U.S. during the COVID-19 pandemic [3]. Declines in the incidence of other bacterial and non-SARS-CoV-2 respiratory viral infections during the COVID-19 pandemic were also reported in the U.S. and Canada [13, 14]. These findings also align with reports from England, where pertussis incidence substantially decreased and no SARS-CoV-2 coinfections were identified between July 2020 and June 2021 [12].

Notably, our results suggest a greater proportion of patients with coinfections than with pertussis alone were Hispanic or Latino and Black or African American persons. These results align with research throughout the pandemic reporting a greater burden of COVID-19 among racial and ethnic minority groups in the U.S. [15]. Underlying disparities in chronic illnesses that predispose Black and Hispanic or Latino persons to more severe disease, in addition to inequitable healthcare access and variable quality of care delivered based on racial and ethnic identity, may contribute to disproportionate burden and severity of illness [15].

The increased frequency of seizures, hospitalization, and death observed among patients with coinfections in our analysis suggests that disease among coinfected patients may be more severe. This contrasts with previous studies of pertussis coinfections, which report that coinfections between *B*. *pertussis* and other respiratory viral infections are common but not associated with increased clinical severity [16, 17]. One possible reason for this difference could be that previous studies of *B*. *pertussis* and respiratory viral coinfections have been conducted among hospitalized patients only, limiting their ability to observe clinical severity outcomes across the disease spectrum and in non-hospitalized patients. Although our results represent a small number of pertussis and SARS-CoV-2 coinfections, increased clinical severity of bacterial and viral coinfections have been documented for other respiratory pathogens [6].

Our study has some limitations. There have been changes in pertussis and SARS-CoV-2 testing throughout the pandemic, including the availability of at-home antigen tests and telehealth visits for SARS-CoV-2, and an increased use of respiratory panel testing. Similarly, testing for both pathogens was performed at clinicians’ discretion; clinicians may be less likely to test for pertussis following a positive SARS-CoV-2 result and vice versa, which could lead to under ascertainment of coinfections. Additionally, while the burden of pertussis is greatest among infants [4], the burden of SARS-CoV-2 during the pandemic was greatest in older age groups [5]. Therefore, underdiagnosis of pertussis in older age groups could lead to under ascertainment of coinfections among this age group. There is also potential for misclassification among probable pertussis cases since these are not laboratory confirmed. Although missing COVID-19 vaccination data is also a limitation, we would not expect any direct cross-protection on pertussis infection from COVID-19 vaccines and vaccine availability and eligibility changed over the course of the study period. Finally, there is not an established coinfection definition; in this analysis we used a 30-day window in line with previous literature [12]. It is known that PCR positivity for SARS-CoV-2 can persist beyond the acute phase of infection, potentially detecting non-viable virus; therefore, future work could examine the sensitivity of this time frame. Given small numbers we did not conduct significance testing; larger studies are needed to further investigate potential associations between coinfection and disease severity and interrogate potential differences in outcomes by SARS-CoV-2 variants.

Despite the decline in reported pertussis during the COVID-19 pandemic, the data presented in this study is important for public health practitioners and clinicians as it highlights that pertussis coinfections with SARS-CoV-2 do occur and patients are more likely to report severe complications and outcomes. Understanding this relationship is important for healthcare providers to prioritize and manage high-risk populations effectively. Continued monitoring of pertussis incidence alongside respiratory viral infections will be important to ensure integrated management strategies for respiratory infections as the pertussis burden returns to pre-pandemic levels.

## Supporting information

S1 Text Pertussis case definition. (DOCX)

S1 Fig Flow chart of pertussis patients included in analytic sample. (TIF)

S2 Fig Temporality of SARS-CoV-2 infections detected among pertussis patients, 2020–2023 (n = 71). (TIF)

S1 Table Characteristics of patients with pertussis and SARS-CoV-2 coinfections, 2020–2023. (DOCX)
