# Supplementary material for: SARS-CoV-2 coinfections among pertussis cases identified through the Enhanced Pertussis Surveillance system in the United States, January 2020–February 2023
Source: PLoS One. 2024 Dec 4;19(12):e0311488. doi: 10.1371/journal.pone.0311488 (PMC11616843; doi:10.1371/journal.pone.0311488)
Supplement: S1 Text — (DOCX) [file pone.0311488.s001.docx]

**S1 Text**. Pertussis case definition^[[1]](#footnote-1)^

**Probable**

- In the absence of a more likely diagnosis, illness meeting the clinical criteria^[[2]](#footnote-2)^

OR

- Illness with cough of any duration, with
  - At least one of the following signs or symptoms:
    - Paroxysms of coughing; or
    - Inspiratory whoop; or
    - Post-tussive vomiting; or
    - Apnea (with or without cyanosis)

AND

- - Contact with a laboratory confirmed^[[3]](#footnote-3)^ case (epidemiologic linkage^[[4]](#footnote-4)^)

**Confirmed**

Acute cough illness of any duration, with

- Isolation of *B. pertussis* from a clinical specimen OR
- PCR positive for *B. pertussis*

1. [Pertussis (Whooping Cough) (Bordetella pertussis) 2020 Case Definition | CDC](https://ndc.services.cdc.gov/case-definitions/pertussis-2020/) [↑](#footnote-ref-1)
2. Clinical criteria: in the absence of a more likely diagnosis, a cough illness lasting ≥2 weeks, with at least one of the following signs or symptoms: paroxysms of coughing; OR inspiratory whoop; OR post-tussive vomiting; OR apnea (with or without cyanosis). [↑](#footnote-ref-2)
3. Confirmed laboratory evidence: isolation of *B. pertussis* from a clinical specimen; positive polymerase chain reaction (PCR) for *B. pertussis*. [↑](#footnote-ref-3)
4. Epidemiologic linkage: contact with a laboratory-confirmed case of pertussis. [↑](#footnote-ref-4)
