# Supplementary figures and images for: SARS-CoV-2 coinfections among pertussis cases identified through the Enhanced Pertussis Surveillance system in the United States, January 2020–February 2023
Source: PLoS One. 2024 Dec 4;19(12):e0311488. doi: 10.1371/journal.pone.0311488 (PMC11616843; doi:10.1371/journal.pone.0311488)

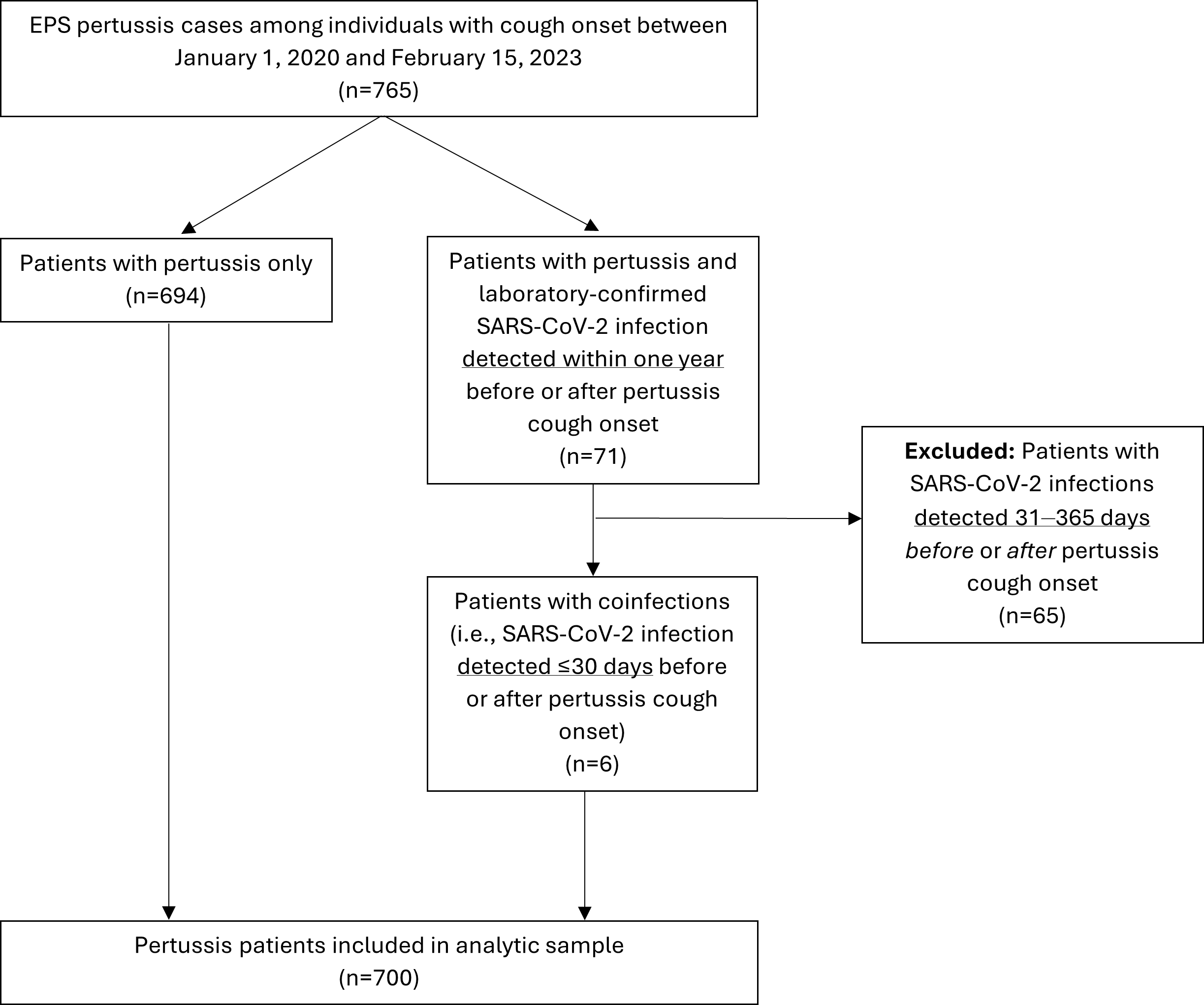

Supplement: S1 Fig — (TIF) [file pone.0311488.s002.tif]

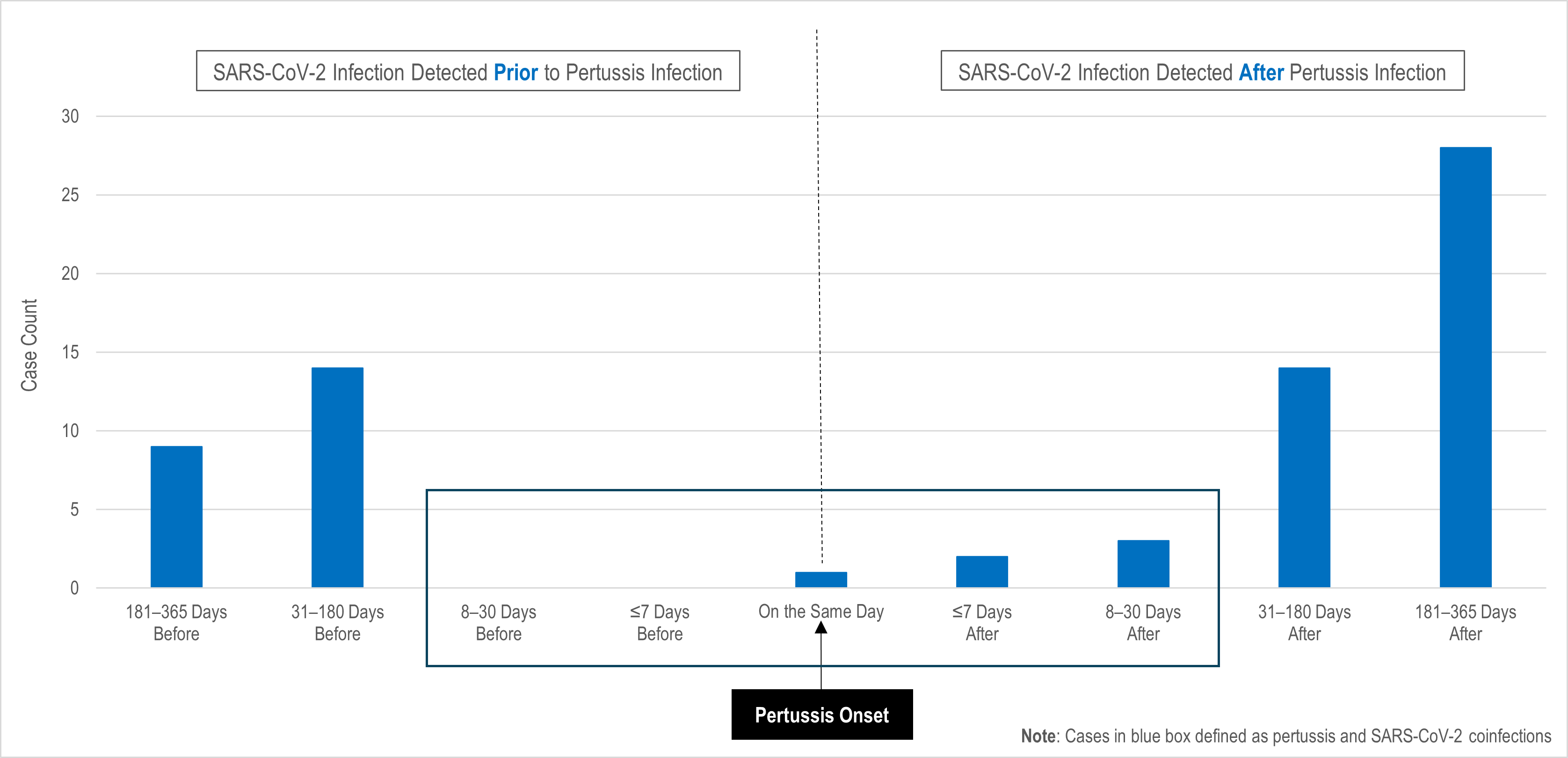

Supplement: S2 Fig — (TIF) [file pone.0311488.s003.tif]
