# Supplementary material for: SARS-CoV-2 coinfections among pertussis cases identified through the Enhanced Pertussis Surveillance system in the United States, January 2020–February 2023
Source: PLoS One. 2024 Dec 4;19(12):e0311488. doi: 10.1371/journal.pone.0311488 (PMC11616843; doi:10.1371/journal.pone.0311488)
Supplement: S1 Table — (DOCX) [file pone.0311488.s004.docx]

**S1 Table.** Characteristics of patients with pertussis and SARS-CoV-2 coinfections, 2020-2023

|  | **Patient 1** | **Patient 2** | **Patient 3** | **Patient 4** | **Patient 5** | **Patient 6** |
| --- | --- | --- | --- | --- | --- | --- |
| **Age (years)** | <1 | ≥20 | ≥20 | <1 | ≥20 | ≥20 |
| **Pertussis Immunization** | Unvaccinated | Insufficiently vaccinated | Unknown | Unvaccinated | Up to date | Up to date |
| **Maternal History of Tdap**^1^ | ✓ | n/a | n/a | ✓ | n/a | n/a |
| **Hospitalized** | ✓ | ✓ | ✓ | -- | -- | -- |
| **ECMO** | -- | -- | -- | n/a | n/a | n/a |
| **Ventilator** | -- | ✓ | -- | n/a | n/a | n/a |
| **Underlying Medical Conditions**^2^ | -- | -- | ✓ | Unknown | ✓ | -- |
| **Note**: n/a, not applicable. ^1^Maternal history of Tdap defined as patient’s mother reporting every received Tdap. ^2^Underlying medical conditions includes: asthma, COPD/emphysema, cerebral palsy, cystic fibrosis, seizure disorder, immunocompromised. | | | | | | |
